# Application of Pulsed Rhythmic Drug Administration to Ovulation Induction Therapy in PCOS Patients with Clomiphene-Resistance: a Retrospective Research

**DOI:** 10.1007/s43032-021-00639-7

**Published:** 2021-06-03

**Authors:** Xinyue Zhang, Aiyan Zheng, Jihong Yang, Ting Feng, Yan Zhang, Yingying Hao, Suying Li, Yun Qian

**Affiliations:** 1 Reproductive Medical Center of the Second Affiliated Hospital of Nanjing Medical University, Nanjing, 210011 China; 2 Center for Reproduction and Genetics, Suzhou Municipal Hospital, the Affiliated Suzhou Hospital of Nanjing Medical University, Suzhou, 215002 China

**Keywords:** Pulsed rhythmic administration, Polycystic ovary syndrome, Intrauterine insemination

## Abstract

There is currently a dispute over the choice of ovulation induction treatment for infertile women with polycystic ovary syndrome (PCOS). The objective of this study is to compare the therapeutic effect of pulsed rhythmic administration protocol (PRAP) with conventional letrozole + human menopausal gonadotropin (HMG) in patients with clomiphene-resistance polycystic ovary syndrome (PCOS). A retrospective analysis of 821 intrauterine insemination (IUI) cycles between January 2015 and January 2020 was performed. Of these, 483 cycles were treated with a pulsed rhythmic administration protocol (PRAP), and 338 cycles were treated with conventional letrozole + HMG protocol (LHP). The therapeutic effect of the two protocols has been compared. The pregnancy rate was 18.07% in the LHP and 27.07% in the PRAP. The ongoing pregnancy rate in LHP was 14.46% and in PRAP was 22.73%. The research suggests that PRAP is more effective than LHP and could be an adequate ovulation induction strategy for the IUI cycle of patients with clomiphene-resistance PCOS.

## Introduction

Polycystic ovary syndrome (PCOS) is a common endocrine disease in women of childbearing age, and it is the main cause of female infertility worldwide [1]. It may contribute to many adverse reproductive, metabolic, and psychological effects, with unknown etiology, which may be related to environmental factors, genetic conditions, and intrauterine exposure [2]. Polycystic ovary syndrome is characterized by a disturbed menstrual cycle (oligomenorrhea or amenorrhea), elevated androgen levels (hyperandrogenism), and multiple ovarian cysts (polycystic ovary) [3]. Currently, there is no cure for PCOS, and the treatment focuses on relieving symptoms and emphasizing a healthy lifestyle to minimize the risk of complications [4].

Intrauterine insemination (IUI) is widely used to improve the chances of pregnancy. It is simple, easy to manage, and relatively low cost, especially for patients with unexplained or mild male infertility [5]. Lifestyle change such as exercise and weight loss in overweight women is considered the first treatment for women with PCOS [6]. However, in the reproductive field, infertility caused by chronic anovulation is the most common reason for women with PCOS to seek treatment [7]. Among these women, ovulation induction is the better choice; thus, CC used to be the first-line treatment; letrozole should be the first-line treatment according to new guidelines [8–10]. However, clinical evidence indicates that although the ovulation rate for patients is higher after ovulation induction, the clinical pregnancy rate is not satisfactory [11]. At the same time, ~20% women with PCOS are resistant to CC, they remains anovulatory under CC induction after three cycles, and they could be offered the therapy of low-dose gonadotropin [8]. Simultaneous implementation of ovulation induction and IUI is one of the most common solutions. Gonadotropins are used in IUI [12]; however, the use of exogenous gonadotropin increases the risk of multiple pregnancies; intense monitoring of ovarian response is necessary [12].

There is currently a dispute over the choice of ovulation induction treatment for infertile women with PCOS. Previous literature has shown that ovulation induction schemes with single or combined drugs have their advantages and disadvantages and are capable of achieving different degrees of clinical efficacy [9, 13–16]. In the stair-step protocol, if the response is not sufficient, the dosage should be increased immediately [17], resulting in a multiple pregnancy rate almost ten times that of natural delivery [18]. In the low-dose step-up protocol, the frequency of extreme ovarian hyperstimulation is 4.6–11.0%, while the multiple pregnancy rate reaches 33% [19]. Xi et al. found that compared with HMG alone, letrozole or CC combined with HMG reduced the HMG dose and the duration of treatment and the letrozole + HMG group achieved the highest rate of monofollicular development and reduced the risks of hyperstimulation for ovarian induction [15]. The final objective for women with PCOS is to achieve unifollicular cycles without causing OHSS and multiple pregnancies [19]. In the long-term clinical practice of the Reproductive Medicine Center of the Second Affiliated Hospital of Nanjing Medical University, pulsed rhythmic administration protocol (PRAP) has been shown to have achieved a successful pregnancy rate in the ovulation induction in infertile PCOS women; they had unsuccessful ovulation induction treatment with CC after three cycles. In this study, we retrospectively analyzed conventional letrozole + human menopausal gonadotropin (HMG) and PRAP to provide a new and appropriate regimen for ovulation induction in patients with clomiphene-resistance PCOS.

## Material and Methods

### Research Subjects

**Fig. 1:**
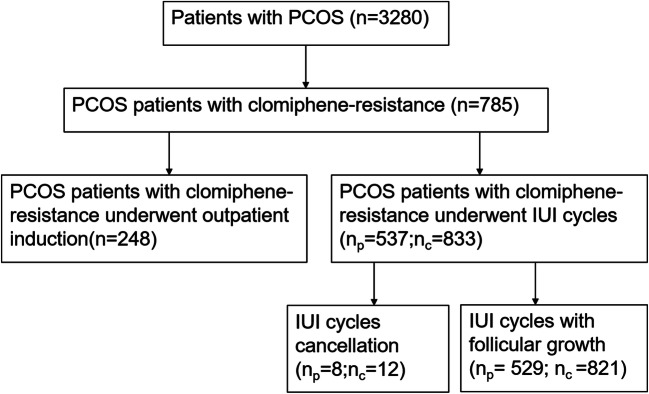
Flow chart of clomiphene-resistance PCOS patients’ inclusion in the Reproductive Medical Center of the Second Affiliated Hospital of Nanjing Medical University. np: number of patients, nc: number of cycles

For retrospective analysis, clomiphene-resistance PCOS patients undergoing IUI-assisted pregnancy at the Reproductive Medicine Center of the Second Affiliated Hospital of Nanjing Medical University from January 2015 to January 2020 were collected. The screening process is shown in detail in Fig. 1. There were 785 PCOS patients with clomiphene-resistance, among which 537 patients underwent 833 IUI cycles, and the others underwent outpatient induction. The cycle cancellation rate was 1.44%. Data were obtained from the medical records received from patients. The study was approved by the ethics committee of the medical institution. Every participant has signed informed consent for the study. All women met the following criteria: (1) According to the Rotterdam Consensus, they have been classified as PCOS [20]; (2) females had been infertile for more than 1 year; (3) females were 21–37 years of age; (4) females had CC resistance after three cycles; (5) females had at least one fallopian tube unobstructed by salpingography or laparoscopy or hysteroscopy; (6) males had at least two semen tests before IUI, excluding azoospermia and severe oligospermia. Exclusion criteria were as follows: (1) the female has a history of other endocrine diseases, such as hyperprolactinemia, thyroid dysfunction or diabetes, tumor history, and family tumor history; (2) B-ultrasonography of the female had intrauterine adhesions, endometrial polyps, and uterine malformations; (3) she has premature ovarian failure; (4) chromosomal abnormalities were present in one or both sides of the couple. After consulting infertility treatment and IUI surgery, induction programs were suggested to all participants. Patients were divided into two groups based on the professional judgment of the experts and the wishes of the patients. The two induction programs have been compared, while the baseline characteristics were similar in both groups.

### Ovarian Induction Programs

#### Letrozole + HMG Protocol (LHP)

Letrozole was taken one to two pills daily from day 3 to day 7 of the menstrual cycle in women treated with LHP (5mg letrozole was used for patients with BMI >40) [21]. Meanwhile, from day 3 of menstruation to the day before human chorionic gonadotropin (hCG) triggering, HMG 75-225IU was administered by injection every other day. Ultrasound examination was performed on day 9 of the menstrual cycle to examine the number and size of follicles and endometrial thickness. From this stage on, the dose of HMG was adjusted according to the individual response of each patient. When one or two follicles were 18 mm in diameter, hCG (10000 IU) was injected intramuscularly to trigger oocyte maturation.

#### Pulsed Rhythmic Administration Protocol (PRAP)

**Fig. 2:**
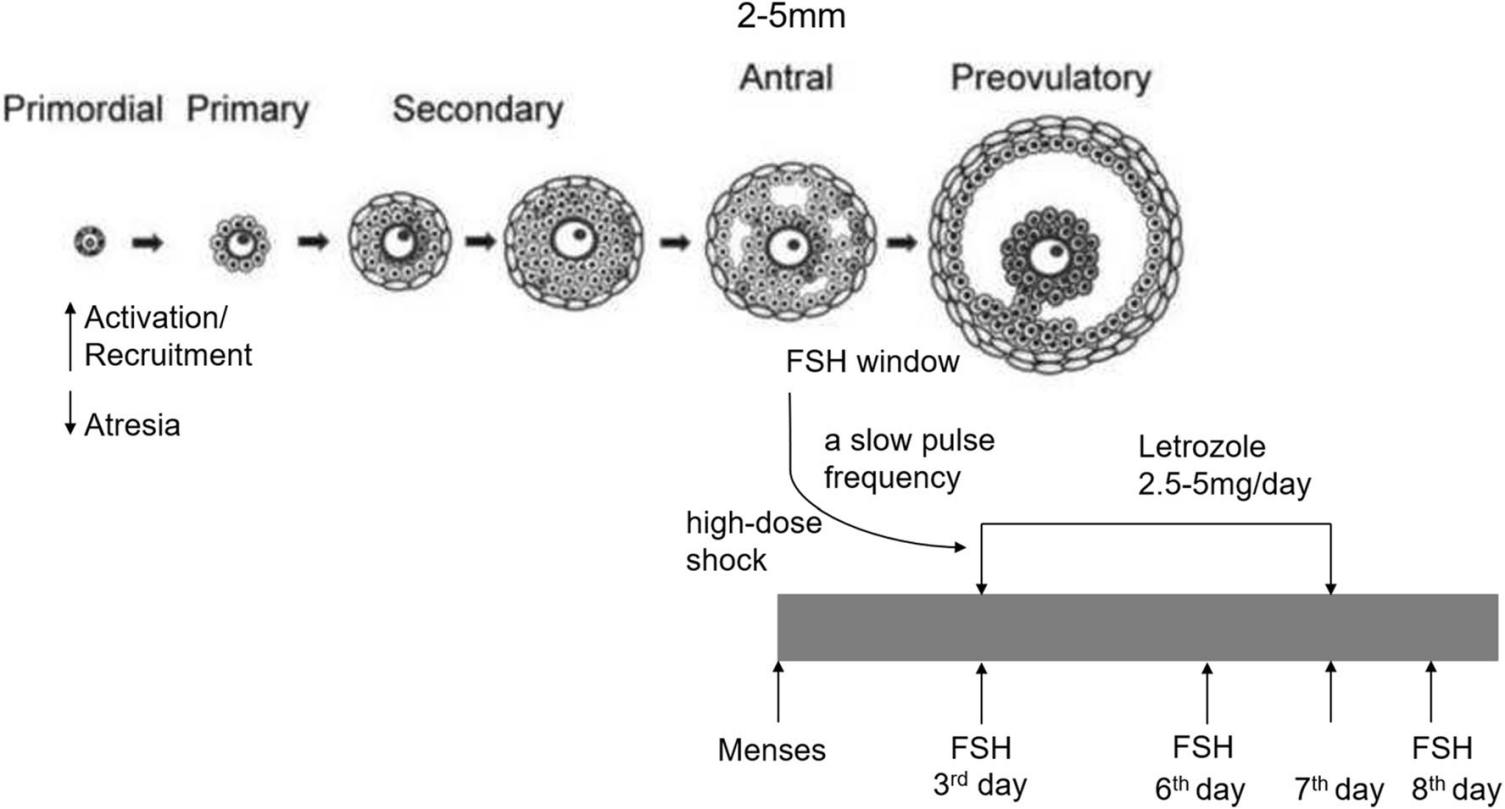
The pulsed rhythmic administration protocol

One or two pills of letrozole were taken every day from day 3 to 7 of the menstrual cycle (5mg letrozole was used for patients with BMI >40) [21]. Meanwhile, follicle-stimulating hormone (FSH) was given on days 3, 6, and 8 of menstruation with an initial dose of 75 IU. Ultrasound examination was performed on day 9. When the follicle diameter was more than 10 mm, the same dose of HMG was used until the follicle matures. If no significant follicular growth was observed on day 9, the dosing interval was shortened, and FSH was changed to injected every other day or daily; without any change in dose, it was still the initial 75IU. When the interval of administration was shortened and the follicle diameter was more than 10 mm, the same dose of HMG was used until the follicle matures. If there was no dominant follicular development in this cycle after the administration interval was shortened and FSH injection was continued for more than day 16, the cycle will be cancelled and the initial dose of FSH in the next cycle will be increased to 150 IU; the interval was maintained with the same pattern of administration rhythm. If there was still no dominant follicular development, the initial dose of FSH in the next cycle will be increased to 225 IU (this is the maximum dose). The detail process is shown in Fig. 2.

### Intrauterine Insemination (IUI) Technique

Semen samples were collected according to WHO standards, while abstinence was needed for 3–5 days before IUI was performed. Semen was collected into a sterile semen cup by a masturbation and liquefied at room temperature for 30 min. The semen was treated with a gradient centrifugation method, and routine examinations were carried out before and after treatment. If the total amount of the male forward motile sperm was not less than 10 × 10^6^/ml after the procedure, the treated semen was slowly injected into the woman’s uterine cavity along the direction of the cervix, and the female rested for 30 min after the operation.

### Luteal Support and Postoperative Follow-Up

Luteal support was given after ovulation by oral administration of dydrogesterone 40 mg/day for 14 days. After 14 days, blood or urine HCG was measured. If urine HCG was positive or blood HCG ≥ 50 mIU/ml, it was identified as biochemical pregnancy. If the pregnancy sac was found by transvaginal ultrasound, it was diagnosed as a clinical pregnancy. The ongoing pregnancy rate was defined as the percentage of women with ongoing pregnancy ≥12 weeks [22].

### Statistical Analysis

SPSS 18.0 software (SPSS, USA) has been used for statistical analysis. The data were expressed as mean ± standard deviation and analyzed by t-test (The data were normally distributed after analyzed.). The ovulation rate and pregnancy outcome were analyzed by the chi-square test or Fisher exact test. P < 0.05 was statistically significant.

## Results

The number of ovulation cycles was 249 in the LHP group, and there were 374 ovulation cycles in the PRAP group. The ovulation rate in the LHP group was 77.43% and in the PRAP group was 73.67%; there were no differences between the two groups (P=0.215). Of the 623 ovulation cycles, there were 374 cycles and 211 participants in the PRAP group and 249 cycles and 141 participants in the LHP group, with no moderate or severe ovarian hyperstimulation. Both groups had relatively many patients with primary infertility. There were four patients in the PRAP group and two patients in the LHP group used letrozole in a dose of 5mg. The baseline characteristics of patients in the two groups are shown in Table 1. The mean age of female patients in the PRAP group was 27.71±3.10 years, and that of the LHP group was 27.39±3.38 years. There was no significant difference in female age, male age, and infertility duration. Body mass index (BMI) was 25.07±4.51 in the PRAP group and 25.21±4.55 in the LHP group. The basal antral follicle count (AFC) was 25.14 ± 6.97 in the PRAP group and 24.66 ± 6.82 in the LHP group. The baseline characteristics of both groups were similar, which means that the two sets of data were comparable.

**Table 1:** Baseline characteristics of the letrozole + HMG protocol (LHP) and the pulsed rhythmic administration protocol (PRAP)

Parameters	LHP	PRAP	P
Number of cycles	338	483	NA
Rate of ovulation (%)	73.67 (249/338)	77.43 (374/483)	0.215
Number of participants	141	211	NA
Primary infertility	182	284	NA
Secondary infertility	67	90	NA
Age, female, years	27.39±3.38	27.71±3.10	0.235
Age, male, years	28.80±3.85	28.82±3.30	0.960
Duration of infertility years	2.90±1.79	2.80±1.78	0.523
BMI	25.21±4.55	25.07±4.51	0.697
Basal AFC	24.66±6.82	25.14±6.97	0.394

Data are expressed as mean ± SD. P<0.05 significantly different from control group

Table 2 shows the levels of essential sex hormones. Serum FSH was 6.54 ± 2.22 in the PRAP group and 6.61 ± 1.82 in the LHP group. Meanwhile, serum luteinizing hormone (LH) was 5.06 ± 2.95 in the PRAP group while 6.41 ± 3.27 in the LHP group. The serum LH level in each group was lower than that of FSH, and there was no difference in serum testosterone levels, suggesting that the endocrine status of clomiphene-resistance PCOS patients was normal and suitable for ovulation induction therapy. There was no significant difference in serum estradiol, prolactin (PRL), and progesterone levels.

**Table 2:** Basal hormone levels between the letrozole + HMG protocol (LHP) and the pulsed rhythmic administration protocol (PRAP)

Parameters	LHP	PRAP	P
Serum FSH	6.61±1.82	6.54±2.22	0.761
Serum LH	6.41±3.27	5.06±2.95	0.000
Serum E2	47.75±27.54	54.87±64.57	0.294
Serum T	0.57±0.24	0.56±0.22	0.527
Serum PRL	11.69±5.15	12.35±5.72	0.532
Serum P	0.62±0.18	0.89±0.53	0.134

Data are expressed as mean ± SD. P<0.05 significantly different from control group

As shown in Table 3, the total dose of FSH/LH in the PRAP group was 953.24 ± 483.22IU, and that in the LHP group was 798.39 ± 559.35 IU. The duration of induction in the PRAP group was 5.39 ± 2.00 days, which was shorter than that in the LHP group. The final cost of LHP group was 804.13±823.49, and the PRAP group was 887.83±469.74 (P=0.147). There was no significant difference between the two groups in the number of dominant follicles and endometrial thickness on the day before IUI surgery. Neither group had moderate or severe ovarian hyperstimulation. Biochemical pregnancy and clinical pregnancy in the PRAP group were higher than those in the LHP group. Among pregnant women, there were eighteen twin pregnancies in the PRAP group and six twin pregnancies in the LHP group. There was one case of ectopic pregnancy in the LHP group. However, four patients in the PRAP group underwent multiple pregnancy reduction. There was no significant difference in the abortion rate between the two groups. The ongoing pregnancy rate in the PRAP group was higher than that in the LHP group.

**Table 3:** Clinical characteristics of the letrozole + HMG protocol (LHP) and the pulsed rhythmic administration protocol (PRAP)

Parameters	LHP	PRAP	P
Total dose of FSH/LH (IU)	798.39±559.35	953.24±483.22	0.000
Duration of induction (days)	7.75±4.63	5.39±2.00	0.000
The final cost (RMB)	804.13±823.49	887.83±469.74	0.147
Number of dominant follicles	1.20±0.47	1.18±0.50	0.540
Endometrial thickness on the day before IUI (cm)	9.09±2.32	9.11±2.00	0.908
The incidence of OHSS (%)	0	0	NA
Biochemical pregnancy (%)	54 (21.67)	110 (29.41)	0.032
Clinical pregnancy (%)	45 (18.07)	101 (27.01)	0.010
Twin pregnancies (%)	6 (2.19)	18 (4.81)	0.127
Miscarriage (%)	9 (3.61)	16 (4.28)	0.679
Ongoing pregnancy ≥12 weeks, no. of women (%)	36 (14.46)	85 (22.73)	0.011

Data are expressed as mean ± SD. P<0.05 significantly different from control group

## Discussion

### Main Results

Present retrospective analysis suggests that PRAP may be a useful stimulus protocol for women with clomiphene-resistance PCOS. On the basis of not significantly increasing the final cost of treatment, the ongoing pregnancy rates in the PRAP group have increased significantly relative to the LHP group.

### Interpretation of Results

In women with PCOS, pulsation of gonadotropin-releasing hormone (GnRH) is usually increased, resulting in an increase in the LH released by the pituitary gland and the LH/FSH ratio [18]. PCOS-associated ovarian defects are affected by various internal and external ovarian factors, which affect follicular development in multiple stages, causing increased recruitment, failure to gain dominance, or atresia, leading to antral follicular developmental stagnation [23]. Most of the phenotypes of anovulatory PCOS are associated with the 2–8 mm antral follicles accumulation [24]. Since patients with PCOS typically have high LH and low FSH levels, estrogen receptor antagonists or aromatase inhibitors are used to reduce the negative feedback of estrogen on pituitary FSH secretion, or exogenous FSH is used to assist ovulation [25]. Therefore, letrozole and exogenous FSH were used to promote ovulation in both the schemes in this study.

During adolescence, the maturation of the hypothalamus-pituitary-gonad (HPG) axis contributes to the pulsed release of FSH and LH in the pituitary gland, resulting in the development of antral follicular cycle ≥ 2 mm, and the initiation of ovulation and menstrual cycle [26]. FSH is a vital nutrient factor affecting follicular cell proliferation, survival, and periodic recruitment of antral follicles. Follicular development can be divided into gonadotropin-dependent and gonadotropin-reactive stages. Exogenous gonadotropin therapy is likely to promote the growth of large antral follicles and smaller preantral follicles [27]. During the physiological human menstrual cycle, a slight increase in serum FSH level (~ 30%) is enough to initiate the growth of the most sensitive ovarian follicles, because the negative feedback of estradiol is reduced throughout the follicular period [28]. Along with follicular growth, they produce estradiol, which provides negative feedback on FSH secretion, thereby limiting the “FSH window”—the period during which FSH levels are held above the threshold to prevent non-dominant follicles atresia [29]. There is differential sensitivity between individual antral follicles in the cohort, and single follicle growth can be successfully induced in the ovulation induction process by gradually reaching the lowest threshold of the most sensitive follicle [30].

In hyperandrogenemia women destined to develop PCOS, this nocturnal increase in ovarian steroids may not be sufficient to inhibit the GnRH pulse generators, resulting in sustained and rapid release of LH, impaired FSH production, and follicular dysplasia. In vivo studies have shown that the faster the frequency of pulsatile exogenous GnRH, the more favorable to the release of LH. Nevertheless, the slow pulse rate was beneficial to the secretion of FSH, which increased serum FSH and decreased serum LH [31]. Therefore, in this research, in the PRAP, FSH was administered on days 3, 6, and 8 of menstruation instead of every other day to facilitate the establishment of a slow pulse frequency and facilitate the secretion of FSH. The role of FSH in follicular development is limited to the early stages, including follicular recruitment and selection. FSH levels in early follicles play an essential role in the final stages of follicular development in the human menstrual cycle. Controlled recruitment of dominant follicles can be achieved by inducing high levels of FSH in the blood within a short period of time during the early stages of the follicular phase [32]. In this study, PRAP had a shorter duration of induction and a higher dose of FSH/LH, as compared to LHP. At the early stage of follicular development, the dominant follicles were established by the high-dose shock. Dominant follicles must remain unique responsiveness and escape the consequences of FSH suppression induced by their own accelerated of estrogen production. The proliferation rate of the granules exceeded its cohort, which makes the dominant follicle to have a higher content of the FSH receptor. On day 9 of the cycle, after ablation of dominant follicles, no other follicles remain fully developed to replace and enable ovulation to occur on time [33]. When the dominant follicle reaches a diameter of 10 mm, divergence occurs [26]. Once the follicle diameter exceeds 10 mm, LH may stimulate the function of granulocyte, and during the selection process, the dependence of dominant follicles on FSH decreases, and the response to LH increases [34]. Therefore, in PRAP, when the follicle diameter is greater than 10 mm, the drug is replaced by the same dose of HMG. PRAP improves both clinical and ongoing pregnancy rates.

### Advantages and Limitations

The advantage of this study lies in its larger sample size compared with other similar studies, and we proposed a new IUI induction protocol for patients with clomiphene-resistance PCOS that may have a significant reference. This study has also observed a few limitations. This is a retrospective study with a short duration and limited types of induction protocols. For patients with clomiphene-resistance PCOS, the ideal treatment has not been determined. Also, the allocation of the subjects was based on the patient’s wishes or the suggestion of the assistant doctor, which allowed for the introduction of biases that may confound the analysis. Therefore, well-designed prospective randomized controlled clinical trials are needed in the future to compare various ovarian induction protocols further.

## Conclusion

PRAP may be the right choice for clomiphene-resistance PCOS patients undergoing the IUI cycle. On the basis of not significantly increasing the final cost of treatment, it can obtain a higher pregnancy rate and ongoing pregnancy rate. However, further prospective clinical trials are required to provide more substantial evidence.

## Data Availability

The datasets used and/or analyzed during the current study are available from the corresponding author on reasonable request.

## Abbreviations

HMG: Human menopausal gonadotropin
PCOS: Polycystic ovary syndrome
IUI: Intrauterine insemination
PRAP: Pulsed rhythmic administration protocol
LHP: Letrozole + HMG protocol
COC: Combined oral contraceptive
hCG: human Chorionic gonadotropin
FSH: Follicle-stimulating hormone
BMI: Body mass index
AFC: Antral follicle count
LH: Luteinizing hormone
PRL: prolactin
GnRH: Gonadotropin-releasing hormone
HPG: Hypothalamus-pituitary-gonad
